# Local persistence of Mann’s soft-haired mouse *Abrothrix manni* (Rodentia, Sigmodontinae) during Quaternary glaciations in southern Chile

**DOI:** 10.7717/peerj.6130

**Published:** 2018-12-18

**Authors:** Lourdes Valdez, Guilermo D’Elía

**Affiliations:** Instituto de Ciencias Ambientales y Evolutivas, Facultad de Ciencias, Universidad Austral de Chile, Valdivia, Chile

**Keywords:** Lowland coastal refugium, Pleistocene, Patagonia, Phylogeography, Historical demography

## Abstract

Quaternary climatic oscillations have impacted Patagonian sigmodontine fauna, leaving traceable genetic footprints. In southern Chile, changes in the landscape included transitions to different vegetation formations as well as the extension of ice sheets. In this study, we focus on the Valdivian forest endemic and recently described sigmodontine species *Abrothrix manni*. We aim to assess the genetic structure of this species, testing for the existence of intraspecific lineages, and inferring the recent demographic history of the species. Analyses were based on the first 801 bp of the mitochondrial gene Cytocrhome-b from 49 individuals of *A. manni* collected at 10 localities that covers most part of its geographic distribution. Genealogical analyses recovered two main intraspecific lineages that are geographically segregated and present an intermediate site of secondary contact. Historical demography shows signal of recent population decrease. Based on these results, we proposed that current genetic diversity of *A. manni* differentiated in at least two distinct refugial areas in southern Chile. This scenario, in addition to be unique among those uncovered for the so far studied Valdivian forest rodents, is noteworthy because of the reduced geographic scale inhabited by the species.

## Introduction

**Figure 1:**
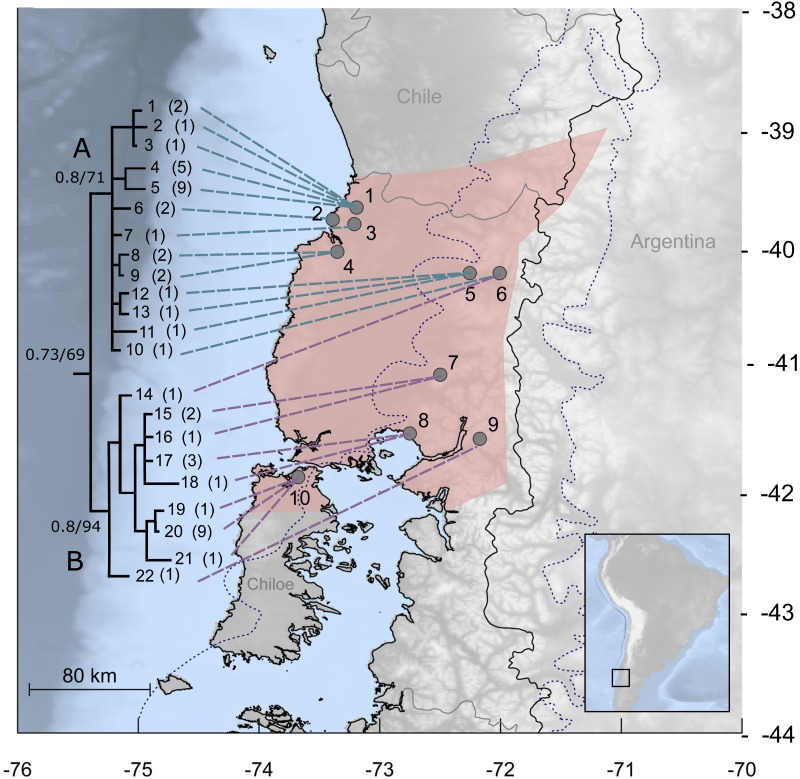
Study area for *Abrothrix manni*. Map of south-central Chile and neighboring areas of Argentina depicting in pink the known distribution of *Abrothrix manni* (taken from D’Elía et al., 2015). Collecting localities of the specimens analyzed here are depicted with dots and numbered as in Table 1. The dotted line indicates the area covered by the ice sheet during the LGM (sensu McCulloch et al., 2000). The tree to the left depicts the genealogical relationships, reconstructed via Bayesian inference, of 22 *Cytb* haplotypic classes of *Abrothrix manni*. Support values correspond to posterior probability and bootstrap proportion in a Maximum Likelihood analysis; number of specimens sharing each haplotype is given in parenthesis.

The southern cone of South America has been the setting of major climatic, tectonic and volcanic activity during the past several million years (Heusser, 1984; Rabassa, Heusser & Rutter, 1990; McCulloch et al., 2000). The Quaternary period was marked by a complex sequence of glacial advances and retreats that dramatically altered Patagonian landscapes (Clapperton, 1994; McCulloch et al., 2000; Hulton et al., 2002). A “Great Patagonian Glaciation” (GPG) occurred approximately 1 MYA, followed by three major post-GPG glaciations during the Early and Middle Pleistocene. The most recent of these events was the Last Glacial Maximum (LGM), which began ∼20,000–18,000 YA and finished 14,000–10,000 YA (Coronato, Martínez & Rabassa, 2004; Ponce et al., 2011). During the LGM, an ice sheet 1,800 km long built up along the axis of the southern Andes (McCulloch et al., 2000). In Southern Chile (south of 39°S) the ice sheet extended toward lowlands covering the eastern half of Los Ríos and Los Lagos Regions, including the south-east of Chiloe Island (Fig. 1). Biotic evidence, including pollen records and beetle remains, indicate that the vegetation type during the last millennia changed from Subantarctic parkland to woodland and Patagonian evergreen forests about 14,000–13,000 YA (Heusser et al., 1996; Moreno et al., 2001). Climatic events of this magnitude affected local abundance and distributional patterns of the local biota leaving traceable genetic footprints in several groups of organisms, which in turn allows identifying refugial areas, i.e., those of population persistence during Pleistocene glaciations, e.g., in trees (Premoli, Mathiasen & Kitzberger, 2010; Mathiasen & Premoli, 2010; Souto et al., 2015), fishes (Zemlak et al., 2008), crustaceans (Xu et al., 2009), frogs (Nuñez et al., 2011), lizards (Vera-Escalona et al., 2012; Muñoz Mendoza et al., 2017).

Patagonian species have been differentially impacted by past climatic dynamics. Different demographic processes and trajectories (e.g., demographic or range expansion or stability, fragmentation, secondary contact) have been recorded in co-distributed species (Lessa, D’Elía & Pardiñas, 2010; Sersic et al., 2011). A pattern of north-to-south postglacial colonization was commonly observed in Patagonian rodents (e.g., *Eligmodontia morgani* and *Reithrodon auritus*), although more complex processes were also reported, such as within-region differentiation and population persistence at putative refugia (Cañón et al., 2010; Lessa, D’Elía & Pardiñas, 2010). Given this rather species-specific response to climatic oscillation in southern Chile and Argentina, it is of interest to explore phylogeographical patterns in additional lineages before any generalization be advanced about the effect of glaciations on the local fauna.

In this study, we focus on the recently described sigmodontine species *Abrothrix manni* (D’Elía, Pardiñas & Teta, 2015). Mann’s soft-haired mouse mainly distributes in the humid forests of north-western Patagonia, in the Chilean Regions of Los Ríos and Los Lagos, including northern Chiloe Island. From west to east, this species is present in the Pacific coast and reaches the Andes; it is also known from a single locality in Neuquén, Argentina (D’Elía, Pardiñas & Teta, 2015; Fig. 1). Because *A. manni* was only recently described, information on its natural history is scarce as no ecological study has focused on it. Much of the available information on the life history of the species has been reported in the literature under *A. sanborni* or of purported hybrids between that species and *A. hirta* (referred earlier as *A. longipilis*; see Teta & Pardiñas, 2014). Mann’s soft-haired mouse is active both day and night (Meserve et al., 1982). It feeds on fungi, moderate amounts of mature arthropods and larvae, plant foliage, seeds, and fruits (Meserve, Lang & Patterson, 1988). *A. manni* breeds through the spring and summer (Meserve et al., 1982). Recording localities of *A. manni*, all laying in the temperate Valdivian rainforest, suggest that the species is a forest specialist.

Compared with most Patagonian mouse species, the geographic range of *A. manni* is noticeably smaller (e.g., Lessa, D’Elía & Pardiñas, 2010; D’Elía, Pardiñas & Teta, 2015) and lies in an area that was highly impacted during the Quaternary period. Here, historical environment diversity ranges from more or less continuous ice cap to putative refugial areas for local biota. A lowland glacial refugium has been proposed along the coast north of the latitude 42°S (Victoriano et al., 2008; Quiroga & Premoli, 2010; Sersic et al., 2011). Other co-distributed (at least partially) sigmodontine species in this area exhibit contrasting genetic footprints. For instance, within the area of sympatry with *Abrothrix manni*, populations of the co-generic *A. olivacea* belong to two distinct intraspecific lineages (Rodríguez-Serrano, Cancino & Palma, 2006), while populations of *Oligoryzomys longicaudatus* are genetically homogeneous, without distinct lineages (Palma et al., 2005; Palma et al., 2012). This is also the case of continental populations of the pudu deer *Pudu puda* (Fuentes-Hurtado et al., 2011). Therefore, given that *A. manni* occurs in a geographic area that was directly affected by Pleistocene glaciations, and considering that the impact of glaciations was differential over species, we address here the phylogeographic pattern of *A. manni.* We aim to assess the species genetic structure, testing for the existence of intraspecific lineages, and inferring past demographic signals.

Theory predicts distinct patterns of genetic variation under distinct demographic and historical scenarios. The literature in this regard is vast (e.g., Tajima, 1989; Slatkin & Hudson, 1991; Fu, 1996; Wakeley & Hey, 1997; Kuhner, Yamato & Felsenstein, 1998) and for a general review we refer the reader to Avise (2000) and Knowles & Maddison (2002).

In this study, we focus on the phylogeographical pattern as revealed by a *Cytb* gene genealogy and on genetic signals of demographic stability/expansion. The main expected signals of the persistence of *A. manni* in Valdivian forest during LGM are: (a) presence of well-supported allopatric clades, (b) multimodal mismatch distributions, (c) significant values of demographic indexes (F, D, hr, and SSD) (d) signals of demographic stability in absolute time calibrated skyline plots. Therewith, we hope to contribute filling the gap in the phylogeographic knowledge of one of the least studied areas of southern South America (Beheregaray, 2008).

## Materials & Methods

### Specimen sampling and data collection

Analyses were based on the first 801 bp of the mitochondrial gene Cytochrome-b (*Cytb*) from 49 individuals of *Abrothrix manni* collected at 10 localities along most part of its known distributional range (see Fig. 1 and Table 1). Fieldwork and procedures with animals were were approved by the Servicio Agricola Ganadero (Permits number 1231/2017, 5611/2013, 2164, 5165) and the Comité de Bioética of the Universidad Austral de Chile (Nb. 311/2018). Nineteen sequences were downloaded from GenBank, while 30 were newly generated from specimens housed at the Colección de Mamíferos de la Universidad Austral de Chile (UACH). Unpublished sequences were generated following the protocol outlined by D’Elía & Pardiñas (2004); external DNA sequencing service was provided by Macrogen (Seoul, South Korea). All new DNA sequences were deposited at GenBank (see accession numbers in Table 1).

**Table 1:** Specimens of *Abrothrix manni* studied with details of the *Cytb* sequence retrieved from each of them. Locality numbers are those depicted in Fig. 1.

#	Locality	Specimen, GenBank accession, haplotype	Reference
1	Región de Los Ríos, San José de la Mariquina, Fundo San Martin; −39.649233, −73.19255	UACH7300, MH917358, 4; UACH7289, MH917347, 4; UACH7291, MH917349, 1; UACH7292, MH917350, 2; UACH7293, MH917351, 1; UACH7294, MH917352, 5; UACH7295, MH917353, 5; UACH7296, MH917354, 5; UACH7297, MH917355, 4; UACH7298, MH917356, 4; UACH7299, MH917357, 5; UACH7301, MH917359, 5; UACH7302, MH917360, 5; UACH7303, MH917361, 5; UACH7304, MH917362, 5; UACH7305, MH917363, 3; UACH7306, MH917364, 5; UACH7879, MH917348, 4	This paper
2	Región de Los Ríos, Valdivia, Curiñanco; −39.7502, −73.390383	UACH7279, MH917366, 6; UACH7307, MH917365, 6	This paper
3	Región de Los Ríos, Valdivia, El Arenal; −39.787367, −73.210909	UACH8016, MH917371, 7	This paper
4	Región de Los Ríos, Corral, Naguilan; −40.016108, −73.351585	UACH7875, MH917367, 8; UACH7876, MH917368, 9; UACH7877, MH917369, 8; UACH7878, MH917370, 9	This paper
5	Región de Los Ríos, Futrono, Cerro Huequecura; −40.192567, −72.254983	UACH7284, MH917342, 12; UACH7285, MH917343, 13; UACH7287, MH917345, 11	This paper
6	Región de Los Ríos, Futrono, Camino Llifen-Maihue, 400 m Oeste de Puente Blanco; −40.193067, −72.0076	UACH7288, MH917346, 10; UACH7286, MH917344, 1	This paper
7	Región de Los Lagos, Parque Nacional Vicente Pérez Rosales, La Picada; −41.033333, −72.5	ER74, GU564046, 15; ER75, GU564047, 1; ER76, GU564048, 1	Palma, Cancino & Rodríguez-Serrano (2010)
8	Región de Los Lagos, Pichiquillaipe, Parque Katalapi; −41.51965, −72.752183	UACH7260, KP665999, 17; UACH7280, KP665998, 1; UACH7281, KP666001, 1; UACH7282, KP666000, 1	D’Elía, Pardiñas & Teta (2015)
9	Región de Los Lagos, Lago Tagua Tagua, Rampa Los Canelos; −41.5643, −72.172217	UACH7283, KP666003, 22	D’Elía, Pardiñas & Teta (2015)
10	Región de Los Lagos, Chiloé, Senda Darwin; −41.883838, −73.663461	ER48, GU564049, 20; ER49, GU564050, 2; ER53, GU564052, 2; ER55, GU564053, 2; ER61, GU564054, 2; ER62, GU564055, 2; ER63, GU564056, 2; ER67, GU564057, 2; ER68, GU564058, 2; ER52, GU564051, 2; UWBM79697, KP666002, 19	Palma, Cancino & Rodríguez-Serrano (2010), D’Elía, Pardiñas & Teta (2015)

### Alignment, molecular diversity, and genealogical reconstruction

Sequences were aligned using Clustal W (Thompson, Higgins & Gibson, 1994) implemented in Mega 7 (Kumar, Stecher & Tamura, 2016) to establish character primary homology. Haplotype and nucleotide diversity indexes were calculated using DNAsp (Rozas et al., 2017). Genetic distances (p-distances) were calculated using Mega 7. Genealogical analyses were based on a matrix containing one representative of each allelic class to speed up searches. Haplotype selection was conducted using DNAsp following a visual corroboration of the absence of segregating sites within haplotype classes. This was conducted observing branch length in a neighbor-joining tree constructed with Mega 7. Sequences of *Cytb* from *Abrothrix andina* (GenBank accession: AF108671), *A. jelskii* (M35714), *A. lanosa* (EU683432), *A. longipilis* (HM167785), *A. sanborni* (KP666004), and *A. olivacea* (HM167800) were used to conform the outgroup. Best-fit model of nucleotide substitution for this dataset was determined based on maximum likelihood estimates of model parameters and Bayesian Information Criterion (BIC) using jModeltest2 (Darriba et al., 2012). The selected model was set in genealogical reconstruction using two approaches, maximum likelihood (ML) and Bayesian inference (BI). The ML tree was inferred using IQ-TREE (Nguyen et al., 2015) implemented in the IQ-TREE web server (Trifinopoulos et al., 2016); branch supports were calculated with the ultrafast bootstrap (Minh, Nguyen & Von Haeseler, 2013). The BI analysis was conducted using MrBayes (Ronquist & Huelsenbeck, 2003); four independent runs were conducted, each consisting of 20 × 10^6^ MCMC iterations, where chains were sampled every 1,000 generations. Convergence among chains was corroborated by inspecting values (<0.01) of the average standard deviation of split frequencies. The first 25% of the samples were discarded as burn-in; remaining trees, sampled well after stationarity was reached, were used to compute a consensus tree with posterior probability (PP) estimates for each clade.

### Genetic structure and historical population analyses

The spatial partition of the observed genetic variation was evaluated using analysis of molecular variance (AMOVA) implemented in Arlequin 3.5 (Excoffier & Lischer, 2010). Several haplotype arrangements were set *a priori* to test specific geographic and genealogical groupings. In a geographic arrangement, sequences were allocated in two groups, one containing northern sampling sites (localities numbers 1 to 6) and another group containing southern sites (localities 7 to 10). In a second arrangement, haplotypes were grouped according to the results of the genealogical analysis (see below). The amount of genetic variation attributable to differentiation among groups (FCT), among localities within groups (FSC), and among localities relative to the total sample (FST) was estimated.

Historical demography was assessed on the basis of the distribution of pair-wise differences and related tests using Arlequin 3.5. A multimodal mismatch distribution indicates that samples were drawn from a population in demographic equilibrium, while a unimodal curve is expected for populations that experienced a recent demographic expansion (Rogers & Harpending, 1992). The smoothness of the mismatch distribution was quantified using the raggedness index (hr); a significant *p*-value indicates that the sample is drawn from a recently expanded population (Harpeding, 1994). The fit of observed data to a null model of stationary population size was tested via the sum of squares deviation (SSD) test. Also, Tajima’s D and Fu’s F were calculated; significant negative values, of both indexes, are indicative of recent population expansion (Tajima, 1989; Fu, 1996) In a different approach, coalescent-base Bayesian skyline plots (Drummond et al., 2005) were also conducted to estimate changes in the effective population size through time, using Beast2 (Bouckaert et al., 2014). Nucleotide substitution and frequencies for each group were estimated from the data; Bayesian Information Criterion was used to choose best-fitting models. The best clock model was selected using Model Selection v1.01 Beast package implemented in BEAST, which was a strict clock. Markov Chain Monte Carlo were let run for 10 M iterations logging trees every 1,000 iterations. Historical demographic plots were constructed using Tracer (Rambaut et al., 2018). Absolute time-scale was calculated using a substitution rate for *Cytb* gene of 0.0031, which was the estimated rate for this gene in the closely related species *Abrothrix longipilis* by Lessa, D’Elía & Pardiñas (2010). Six groups were set to test for signals of demographic change/stability using mismatch distributions, associated demographic tests and Bayesian skyline plots mentioned above. (1) Total sample, which includes the 49 *Cytb* sequences in the dataset; (2) clade A, which considers only the sequences included in this clade (see below); (3) clade B, including only sequences in this clade (see below), (4) northern range, containing all samples from localities 1–6, (5) southern range, with all samples from localities 7–10; and (6) eastern range, containing samples from localities near the Andes, 5–9).

## Results

### Molecular diversity

The 49 sequences of *Cytb* of *Abrothrix manni* contain 46 variable sites that define 22 haplotypic classes (Table 1). Nine haplotypes were found in more than one specimen. No single haplotype was found in more than one collection site; most sites (seven out of 10) exhibited more than one haplotype (Table 1). Overall pairwise distance for the whole dataset is 1.2%. Sequence divergence among localities (Fig. 1) ranged from 0.3% (between localities 3 and 9) to 1.8% (between locality pairs 1–2, 1–5, 1–3, and 2–3). Observed haplotype and nucleotide diversity values for the entire sample are Hd = 0.909 (SD = 0,025) and Pi: 0,01012.

### Gene genealogy

The best model of molecular evolution for the dataset was HKY + G (−lnL = 3499.6757; BIC = 7447.304), which was implemented in both ML and BI analyses. Resulting topologies congruently recover, although without a strong support (Bayesian posterior probability: PP = 0.73; Bootstrap support: BT = 67%) the monophyly of *A. manni.* Haplotypes of *A. manni* form two main clades (Fig. 1). The first one (clade A, hereafter) is composed by 10 haplotypes from 6 localities (1–6) covering the northern part of the study area; support values for this clade are low, PP = 0.8 and BT = 71. The second clade (clade B, hereafter), which is moderately supported (PP = 0.8, BT = 94), includes haplotypes from southern localities (7–10) and one haplotype from a specimen collected at locality 6. Thus, in the area of Llifen (locality 6) both main clades of *A. manni* overlap (Fig. 1). Haplotypes from clades A and B are, on average, 1.7% divergent. Mean divergence within each clade is low; 0.7% and 0.6% for clades A and B, respectively.

### Genetic structure and historical demography

When grouping haplotypes according to a geographical criterion (localities 1–6 vs.7–10), AMOVA shows that 55.6% of the total variation in the data set is attributable to differences between these two geographic groups. The remaining percentage is due to differences among locality sites (21%) and within localities (23.4%). Fixation indexes were significant at these three levels (Table 2). When analyzing the data set grouped according to gene genealogy, the AMOVA shows the same pattern, where the largest fraction of the observed variation is due to differences between groups (clades in this case). Here, a 2% increase was observed in the variation attributable to differences between clades (57.26%) over the mentioned difference between geographic groups (Table 2).

**Table 2:** Analysis of molecular variance. Results of analyses of molecular variance (AMOVA) for two arrangements of samples (for the definition of groups see ‘Materials and Methods’ and Fig. 1). Percentage of variation among groups (AG), among populations within groups (APWG), and within populations (WP) are given.

		Percentage of variation			*F-statistics*		
Grouping criteria	Group content (locality numbers)	AG	APWG	WG	*F*sc	*F*st	*F*ct
Geneaological	G1: specimens from clade A	57.26	20.68	22.06	0.48^*^	0.78^*^	0.57^*^
	G2: specimens form clade B						
Geographic	G1: specimens from northern localities (1–6)	55.61	21.05	23.34	0.47^*^	0.77^*^	0.56^*^
	G2: specimens from Southern localities (7–10)						

**Notes.**

* indicate *p*-values lower than 0.0001.

**Figure 2:**
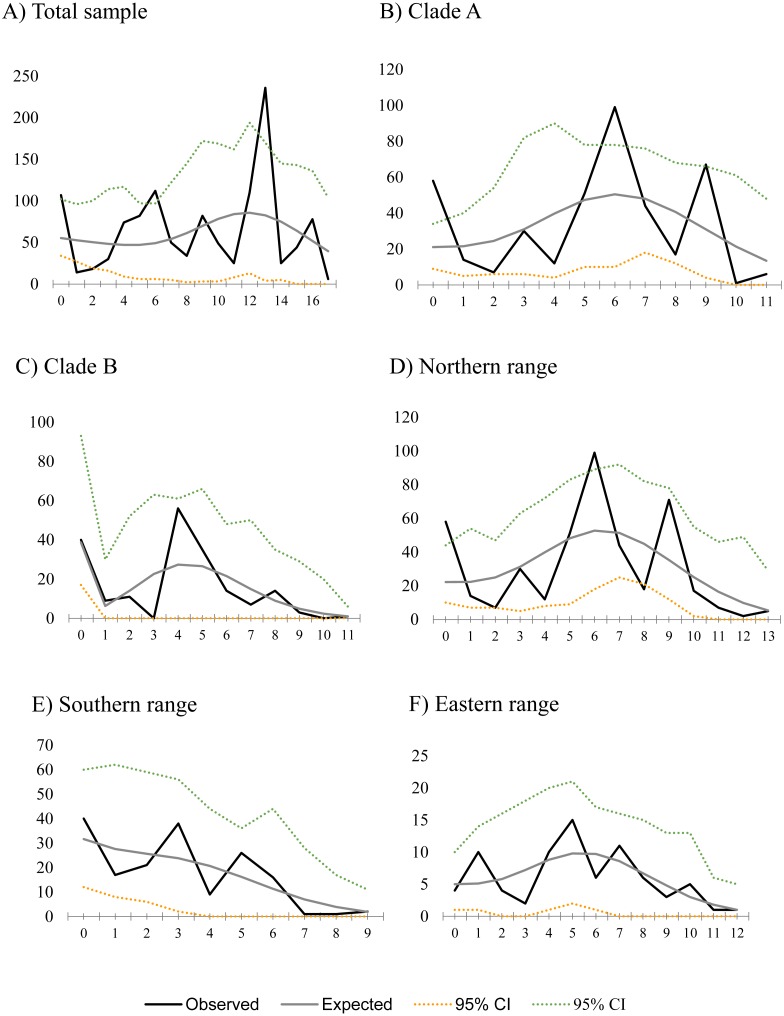
Mismatch distribution for populations of *Abrothrix manni*. Frequency distribution of pair-wise differences among *Cytb* haplotype pairs of *Abrothrix manni* for (A) total sample; (B) clade A; (C) clade B; (D) northern range; (E) Southern range; (F) Eastern range,

**Figure 3:**
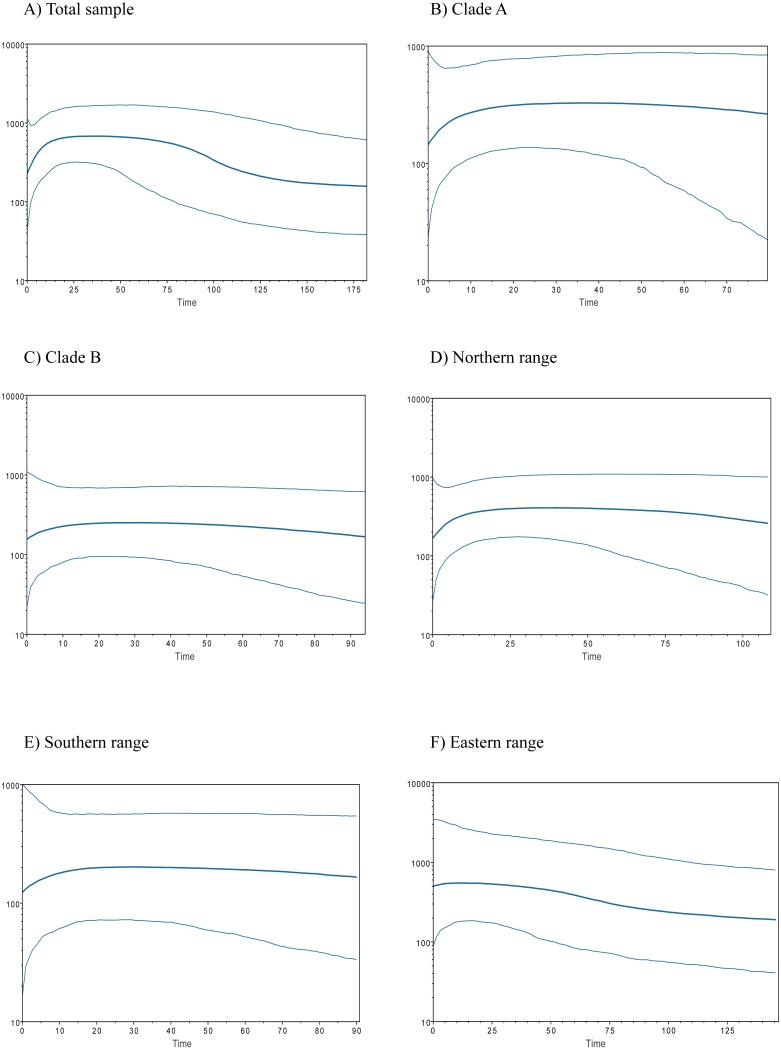
Bayesian skyline plots for populations of *Abrothrix manni*. Bayesian skyline plots showing the change in effective population size (*y*-axis) through time (in thousand years; *x*-axis) for distinct samples of haplotypes of *Abrothrix manni*: (A) total sample; (B) clade A; (C) clade B; (D) northern range; (E) southern range; (F) eastern range.

Historical demography of *A. manni*, assessed by means of four indexes, recovered contrasting signals for six different grouping of samples. When considering the whole dataset, half of the indexes recovered signals of demographic expansion (SSD and Hr), while the rest failed to do so (Tajima’s D and Fu’s Fs; see Table 2). Further exploration of the overall signals led us to calculations per clade and geographic range. Estimations for clade A and northern range exhibited the same pattern as the total sample, with significant values of SSD and Hr (Table 2). On the other hand, calculations for clade B and southern range failed to show signals of population expansion in most tests (Table 2, Fig. 2). Exceptions are marginally significant values of Fu’s F for clade B and highly significant negative Tajima’s D for southern range. Western localities (1–9) are sites that were probably covered by the ice sheet during the last glaciation (see dotted lines in Fig. 1). Demographic tests based on mismatch distribution for this set of localities failed to recover signals of population expansion (Table 3, Fig. 2). Finally, skyline plots helped understanding past demographic dynamics at the light of contradictory signals in demographic tests by showing that after an increase in the effective population size, a more recent decrease have occurred (Fig. 3). This pattern was observed in all groups analyzed, being more evident when analyzing the total sample (Fig. 3). Population size reductions would have taken place during the last 15,000–10,000 years. Skyline plot for the eastern range shows a gradual increment in the population effective size from ca. 100 KYA to 25 KYA and a more recent slight reduction in effective size (Fig. 3F).

## Discussion

Patagonian sigmodontine species have differentially responded to historical climatic events. Many of these broadly distributed species lack significant phylogeographic breaks (Lessa, D’Elía & Pardiñas, 2010). Fewer are the cases where allopatric clades were reported; among these are the Patagonian chinchilla mouse *Euneomys chinchilloides* (Lessa, D’Elía & Pardiñas, 2010), the long-haired mouse *Abrothrix hirta* (Lessa, D’Elía & Pardiñas, 2010) and the olive mouse *Abrothrix olivacea* (Rodríguez-Serrano, Cancino & Palma, 2006; Lessa, D’Elía & Pardiñas, 2010). Notice, however, that these species show phylogeographic structure over their large distributional ranges covering most of the open (the former) or open and forested (the last two) areas of Patagonia. Meanwhile the distributional range of *Abrothrix manni* is considerably smaller than that of the species mentioned above, including the co-generic and co-distributed *A. hirta* and *A. olivacea* (D’Elía, Pardiñas & Teta, 2015). Even so, *A. manni* exhibits a clear differentiation in two main clades that are essentially allopatric (Fig. 1). Interestingly, in our study area, which traditionally has been considered as large refugial area (Villagrán, 1991; Villagrán, Moreno & Villa, 1995) no phylogeographic structure is found in other mammals, such as the olive mouse *A. olivacea* (Rodríguez-Serrano, Cancino & Palma, 2006), the long-tailed mouse *O. longicaudatus* (Palma et al., 2005; Palma et al., 2012), the marsupial *Dromiciops gliroides* (sensu D’Elía, Hurtado & D’Anatro, 2016; clade C in Himes, Gallardo & Kenagy, 2008), and the pudu *Pudu puda* (Fuentes-Hurtado et al., 2011). Therefore, setting aside the limitations of the data analyzed in terms of loci and geographic coverage, the phylogeographic pattern found in *A. manni* is unique among the mammals studied to date in the Patagonian Valdivian forest.

**Table 3:** Demographic history of *Abrothrix manni*. Indexes of demographic history for four arrangements of samples of Abrothrix manni. Tajima’s D (D), Fu’s Fs (Fs), sum of squared deviation (SSD), Harpending’s raggedness index (Hr), and their respective *p*-values to the right. Values in bold are those that support demographic expansion.

	Group content	*D*	*p*	*Fs*	*p*	*SSD*	*p*	*Hr*	*p*
Total sample	Samples from localities 1–10	−0.35	0.42	−1.94	0.28	**0.32**	**0.02**	**0.06**	**0.02**
Clade A	Samples in clade A from localities 1–6	−0.61	0.31	−1.59	0.25	**0.04**	**∼0.00**	**0.10**	**∼0.00**
Clade B	Samples in clade B from localities 6–10	−1.39	0.08	**−22.84**	**∼0.00**	0.06	0.09	0.14	0.06
Northern range	All samples from localities 1–6	−1.02	0.15	−1.92	0.22	**0.03**	**0.02**	**0.08**	**∼0.00**
Southern range	All samples from localities 7–10	**−1.51**	**0.05**	1.27	0.22	0.02	0.47	0.078	0.45
Eastern range	All samples from localities 5–9	−0.86	0.19	−1.74	0.20	0.08	0.08	0.10	0.06

The distributional area of *A. manni* has been highly impacted by the Quaternary climate oscillations. Environmental dynamics in this area include changes in the spatial configuration of forests (e.g., reduction, fragmentation, and expansion) and the extension of a glacial ice cap in pre-Andean and Andean areas during the LGM (Villagrán, Moreno & Villa, 1995; Heusser, Heusser & Lowell, 1999; McCulloch et al., 2000; Ponce et al., 2011). Having in mind that *A. manni* is a forest-dwelling species with a restricted geographic range, the uncovered phylogeographic pattern suggests a process of local differentiation in at least two areas that are latitudinally segregated, one in the north and another in the south of the species distribution. Determining the absolute time for the formation of the two main intraspecific lineages of *A. manni* remains a challenging task. However, considering the estimation of 0.6 MY as the crown age of the species (see Fig. 2 in D’Elía, Pardiñas & Teta, 2015; but note the error bar ranging from more than 1 MY to a few thousand years ago), it is possible that the two main lineages of *A. manni* may have diverged in the context of Pleistocene forest fragmentation occurring in the area (Villagrán, 1991; Villagrán & Hinojosa, 1997; Heusser, Heusser & Lowell, 1999). If, as hypothesized, both main mitochondrial lineages of *A. manni* differentiated in allopatry, it is necessary to propose an event of secondary contact after the differentiation of the northern and southern lineages of *A. manni*. Our sampling design detected a single intermediate locality of sympatry of these lineages (locality 6, Table 1, Fig. 1). Since there are not noteworthy discontinuities in the non-sampled area between the known areas of occupancy of both clades, we anticipate that more sites of contact might be found with a denser geographic coverage.

The existence of Pleistocene refugia in the Valdivian forest of North-Western Patagonia has been proposed in an increasing number of studies, including reptiles (Victoriano et al., 2008; Vera-Escalona et al., 2012; Muñoz Mendoza et al., 2017), amphibians (Nuñez et al., 2011), fishes (Zemlak et al., 2008; González-Wevar et al., 2015) and insects (Xu et al., 2009). An initial model of a single coastal refugium has been rapidly replaced by a more complex scenario of multiple refugia (Premoli, Kitzberger & Veblen, 2000; Premoli et al., 2003; Azpilicueta, Marchelli & Gallo, 2009; Nuñez et al., 2011; Vera-Escalona et al., 2012). In any case, palynological, palaeoclimatic and genetic data suggest that non-glaciated coastal areas from northern Chiloe up northwards were mainly forested during the LGM (Villagrán, 1991; Villagrán & Hinojosa, 1997; Heusser, Heusser & Lowell, 1999). Accordingly, several tree species, such as *Nothofagus pumilio*, *N. oblicua*, *Podocarpus nubigena*, and *Euchryphia cordifolia*, would have persisted not only in the coastal zone, but also near and at Los Andes (Azpilicueta, Marchelli & Gallo, 2009; Quiroga & Premoli, 2010; Mathiasen & Premoli, 2010; Segovia, Pérez & Hinojosa, 2012), some of them probably moving down and uphill during the Pleistocene and the Holocene (Villagrán, 1991; Premoli, Kitzberger & Veblen, 2000). Sersic et al. (2011) identified two types of refugial areas in southern Chile, a peripheral glacial refugium along the western slopes of the Andes, and a lowland glacial refugium located in the Western Chilean Pacific coast. The first one is supported by genetic evidence of—among other plants and animal species—the sigmodontine mouse *Loxodontomys micropus* (Cañón et al., 2010; Sersic et al., 2011). On the other hand, another sigmodontine rodent, the olive mouse *Abrothrix olivacea*, supports the lowland coastal refugium (Smith, Kelt & Patton, 2001). In addition, both coastal and Andean refugia has been also proposed for same species (Premoli et al., 2003; e.g., Xu et al., 2009; Sersic et al., 2011; Nuñez et al., 2011; Vera-Escalona et al., 2012). Whether the suggested refugia for *A. manni* were coastal and/or pre-Andean is uncertain. Both scenarios are plausible for either northern or southern refugium. Irrespective the precise location of refugial areas for northern and southern lineages, our results support the local persistence of *A. manni* during the LGM in its current general distributional area at the Valdivian forest. In line with this idea is the fact that neither demographic indexes nor mismatch distribution test for the six geographical groups of localities showed clear signals of recent population expansion; the eastern range (Table 3) that considers localities that would have been covered by the ice sheet during the LGM (Fig. 1). In addition, the six groups analyzed (Figs. 3A–3E) show a general pattern of population reduction towards the present. Noteworthily, the eastern range group shows a trend of gradual increment in the effective population size between ca. 100 KYA to 25 KYA with a minor reduction towards the present (Fig. 3F). In this regard, it is of interest in future studies to include more samples of *A. manni* from the eastern range, particularly from Argentina (i.e., east side of the Andes) to assess if the uncovered pattern changes.

In addition to the mainland area of Central-Southern Chile, the north-west half of the large Chiloe Island has been also proposed as a refugial area (Villagran, 1988). Due to sampling limitations we could not test here for demographic stability of populations of *A. manni* from Chiloe, but we observed that island genetic variants are closely related to those recovered in the mainland. This was also observed in the marine otter *Lontra felina* (Vianna et al., 2010), the pudu deer *Pudu puda* (Fuentes-Hurtado et al., 2011), the marsupial *Dromicios gliroides* (Himes, Gallardo & Kenagy, 2008), and in the long-tailed mouse *Oligoryzomys longicaudatus* (Palma et al., 2005). These results are in line with well-known evidence showing that during Pleistocene glacial oscillations, the Chiloe Island remained connected with the mainland due to a drop in sea level (Heusser, Heusser & Lowell, 1999).

In north-western Patagonia, different mammals show signals of demographic expansion within our study area. This is the case of *Dromiciops gliroides* (referred as clade C in Himes, Gallardo & Kenagy, 2008), *Abrothrix olivacea* (Smith, Kelt & Patton, 2001; but see Rodríguez-Serrano, Cancino & Palma, 2006) and *Oligoryzomys longicaudatus* (Palma et al., 2012). Contrary to the mentioned species, *A. manni* fails to show signals of recent demographic expansion in both northern and southern portions of its distributional range (Table 3). On the contrary, Bayesian skyline plots showed a temporal line with an increase in the effective population size followed by a reduction towards the present (Fig. 3). This population decline was estimated as occurring about 15,000-10,000 YA. This is the approximate timing of the end of the last glaciation. Distinct processes could explain the inferred recent reduction in population sizes of Mann’s soft-haired mouse. One of this is a scenario where populations of *A. manni* have been negatively affected by interspecific competition with other species, likely sigmodontine rodents, which would have expanded in the area after glacial conditions retreated. Also, it is possible that the demographic reduction experienced by *A. manni* may have been caused by the wildfire that increased in Northern Patagonian forests ca. 11,000–8,500 YA (Abarzúa & Moreno, 2008; Úbeda & Sarricolea, 2016) and/or eruptions such as that of the regional volcanoes Mocho-Choshuenco and Puyehue-Cordón Caulle (Fontijn et al., 2016). For the moment, the understanding of the natural history of *A. manni* is much limited (see D’Elía, Pardiñas & Teta, 2015); as such, the just posed suggestions needs to be further evaluated with additional genetic studies (allowing testing the reduction in population sizes) as well as with field studies.

## Conclusions

The genetic variation of the mitochondrial *Cytb* gene of *Abrothrix manni* supports a history of persistence during Quaternary oscillations in its restricted distributional area at the Valdivian forest of north-western Patagonia. This, however, does not imply that this mouse was not impacted by climatic changes; on the contrary, forest fragmentation would have caused a differentiation process that led to two divergent intraspecific lineages and the posterior secondary contact between them. These conclusions are noteworthy because of the reduced geographic scale inhabited by the species. They also imply that this small area may have harbored multiple Pleistocene refugia.

Finally, with this study, which constitutes the first description of the spatial distribution of the genetic variation and historical demography of the recently described Mann’s soft-haired mouse *A. manni*, we hope to contribute to the understanding of evolutionary history of the local fauna, particularly of the sigmodontine rodents.

## Additional Information and Declarations

### Field Study Permissions

The following information was supplied relating to field study approvals (i.e., approving body and any reference numbers): Animal capture and collection were approved by the Servicio Nacional Ganadero de Chile (SAG) and the study was approved by the Comité de Bioética “Uso de animales en la Investigación”, Universidad Austral de Chile (UACh).

### DNA Deposition

The following information was supplied regarding the deposition of DNA sequences: The Cytochrome-b sequences described here are accessible via GenBank accession numbers MH917342 to MH917371.

### Data Availability

The following information was supplied regarding data availability: The alignment files are available at Dryad: doi:10.5061/dryad.js7r696.
